# Therapeutic preferences and outcomes in newly diagnosed patients with Crohn’s diseases in the biological era in Hungary: a nationwide study based on the National Health Insurance Fund database

**DOI:** 10.1186/s12876-018-0746-6

**Published:** 2018-01-30

**Authors:** Zsuzsanna Kurti, Akos Ilias, Lorant Gonczi, Zsuzsanna Vegh, Petra Fadgyas-Freyler, Gyula Korponay, Petra A. Golovics, Barbara D. Lovasz, Peter L. Lakatos

**Affiliations:** 1 1st Department of Medicine, Semmelweis University, Budapest, H-1083 Hungary; 2 Strategic Analysis Department, National Health Insurance Fund (OEP), Budapest, H-1139 Hungary; 3 Division of Gastroenterology, McGill University Health Centre, 1650 Ave. Cedar, D16.173.1, Montreal, QC H3G 1A4 Canada; 4 Faculty of Health Sciences, Department of Clinical Studies, Semmelweis University, Budapest, H-1088 Hungary; 5 Division of Gastroenterology/Hepatology Unit and Endoscopy, Semmelweis University, Koranyi S 2A, Budapest, H-1083 Hungary

**Keywords:** Treatment strategy, Crohn’s disease, Inflammatory bowel disease, Biological therapy, Surgery, Administrative database, Nationwide

## Abstract

**Background:**

Accelerated treatment strategy, including tight disease control and early aggressive therapy with immunosuppressives (IS) and biological agents have become increasingly common in inflammatory bowel disease (IBD). The aim of the present study was to estimate the early treatment strategy and outcomes in newly diagnosed patients with Crohn’s disease (CD) between 2004 and 2008 and 2009–2015 in the whole IBD population in Hungary based on the administrative database of the National Health Insurance Fund (OEP).

**Methods:**

We used the administrative database of the OEP, the only nationwide state-owned health insurance provider in Hungary. Patients were identified through previously reported algorithms using the ICD-10 codes for CD in the out-, inpatient (medical, surgical) non-primary care records and drug prescription databases between 2004 and 2015. Patients were stratified according to the year of diagnosis and maximum treatment steps during the first 3 years after diagnosis.

**Results:**

A total of 6173 (male/female: 46.12%/53.87%) newly diagnosed CD patients with physician-diagnosed IBD were found in the period of 2004–2015. The use of 5-ASA and steroids remained common in the biological era, while immunosuppressives and biologicals were started earlier and became more frequent among patients diagnosed after 2009. The probability of biological therapy was 2.9%/6.4% and 8.4%/13.7% after 1 and 3 years in patients diagnosed in 2004–2008/2009–2015. The probability of hospitalization in the first 3 years after diagnosis was different before and after 2009, according to the maximal treatment step (overall 55.7%vs. 47.4% (*p* = 0.001), anti-TNF: 73%vs. 66.7% (*p* = 0.103), IS: 64.6% vs. 56.1% (*p* = 0.001), steroid: 44.2%vs. 36.8% (*p* < 0.007), 5-ASA: 32.6% vs. 26.7% *p* = 0.157)). In contrast, surgery rates were not significantly different in patients diagnosed before and after 2009 according to the maximum treatment step (overall 16.0%vs.15.3%(*p* = 0.672) anti-TNF 26.7%vs.27.2% (*p* = 0.993), IS: 24.1%vs22.2% (*p* = 0.565), steroid 8.1%vs.7.9% (*p* = 0.896), 5-ASA 10%vs. 11% (*p* = 0.816)).

**Conclusions:**

IS and biological exposure became more frequent, while hospitalization decreased and surgery remained low but constant during the observation period.

Use of steroids and 5-ASA remained high after 2009. The association between the maximal treatment step and hospitalization/surgery rates suggests that maximal treatment step can be regarded as proxy severity marker in patients with IBD.

## Background

Inflammatory bowel diseases (IBD) are chronic conditions affecting the patient’s quality of life, leading to disability; moreover, a significant proportion of patients require surgery. In the past decades, the prevalence of IBD increased worldwide [1–3]. However, development of medical therapy improved patients’ perspectives significantly. In recent years, treatment strategies and therapeutic targets have evolved: “treat-to-target” has become an important inpatient management and therapeutic goals have been determined [4]. Additionally, accelerated treatment strategy, including tight disease control and early aggressive therapy with immunosuppressives (IS) and biological agents (mostly anti-TNFs) have become increasingly common in IBD. Emerging data suggest that earlier introduction of IS and biologicals may be associated with improved outcomes including lower risk for surgery and hospitalization [5–9]. These improvements in the management of IBD are reflected by clinical guidelines as well [10]. Nevertheless, it is important to investigate how these changing paradigms affect every day clinical practice and clinical outcomes.

The recent ECCO-EpiCom studies based on the 2010 and 2011 inception cohorts provided data about up-to-date IBD incidences, treatment steps and initial disease course of IBD in the biological era in Europe. Differences were reported between Western and Eastern Europe, not only in the prevalence of IBD, but also regarding treatment strategy and disease outcomes [11–13]. Data on the natural history and therapeutic strategies of Crohn’s disease was also reported in population-based studies from North-America, including data from the Mayo clinic, Olmsted Country, Minnesota and the Manitoba Cohort, Canada [8, 14, 15]. It is essential to note, that direct comparison of the above studies may be difficult due to differences in health care insurance systems and data acquisition.

In Hungary, previous population-based studies on the epidemiology and treatment strategy in IBD were reported in the Veszprem population-based IBD database. Nevertheless, data on medical strategy were reported from the pre-biological era [16, 17]. Recently, our group reported nationwide estimate of IBD prevalence from the administrative database of the National Health Insurance Fund [18]. However, the effect of medical therapy on disease outcomes has not been studied yet.

Therefore, the aim of the present study was to investigate the early treatment strategy and outcomes in newly diagnosed patients with Crohn’s disease (CD) between 2004 and 2015 in Hungary. Since the prescription regulations changed significantly at the end of 2008 with easier access to biologicals, it has been aimed to evaluate surgical outcomes and hospitalization rates before and after the change of prescription-regulations in the administrative database of the National Health Insurance Fund (OEP) which is the only (state-owned) health insurance provider in Hungary.

## Methods

We used the administrative database of the National Health Insurance Fund (OEP). Patients were identified through previously reported algorithms using the ICD-10 codes for Crohn’s disease (K50..) in the out- and inpatient (medical, surgical) non-primary care records and drug prescription databases between 1st of January 2004 and 1st of January 2015 [18]. Hospitalizations, surgical episodes and drug prescriptions were identified through the database of the OEP, which contains data on in- and outpatient stays, outpatient procedures and filled drug prescriptions. Any event with a CD-related ICD-10 code was regarded as related.

New patients with Crohn’s disease was defined as no CD-related outpatient visits and/or IBD-related drug prescriptions in the last 3 years before the start of the observational period and at least 2 CD-related outpatient visits and/or IBD-related drug prescription during the observational period. Patients with less than 3 years follow up were excluded from the study. Patients were stratified according to the year of diagnosis and the maximum treatment step during the first 3 years after diagnosis.

We determined time to prescription of different drug classes, (5-ASA, steroid, IS and biologicals) and stratified patient groups according to the maximal therapeutic step (1. group – maximum therapeutic step: 5-ASA therapy (oral and/or topical 5-ASA treatment), 2. group – maximum therapeutic step: steroid therapy (systemic or topical steroids), 3. group – maximum therapeutic step: IS therapy (azathioprine, 6-mercaptopurine, cyclosporine or methotrexate), 4. group – maximum biological therapy (infliximab or adalimumab)). Finally, we calculated hospitalization and surgery rates according to the maximal treatment step and the diagnostic period (diagnosis in 2004–2008 vs. 2009–2015).

### Statistical methods

SPSS® 20 (SPSS Inc., Chicago, IL) was used for statistical analysis. Khi-square test and Fischer-exact tests were used to compare categorical variables. Kaplan-Meier analysis and Cox-Mantel LogRank test was used for time dependent change of the categorical variables. Time from diagnosis were weighted to 30-days periods in the Kaplan-Meier analysis. A *p* value of < 0.05 was regarded as statistically significant.

## Results

### Patient characteristics

**Fig. 1:**
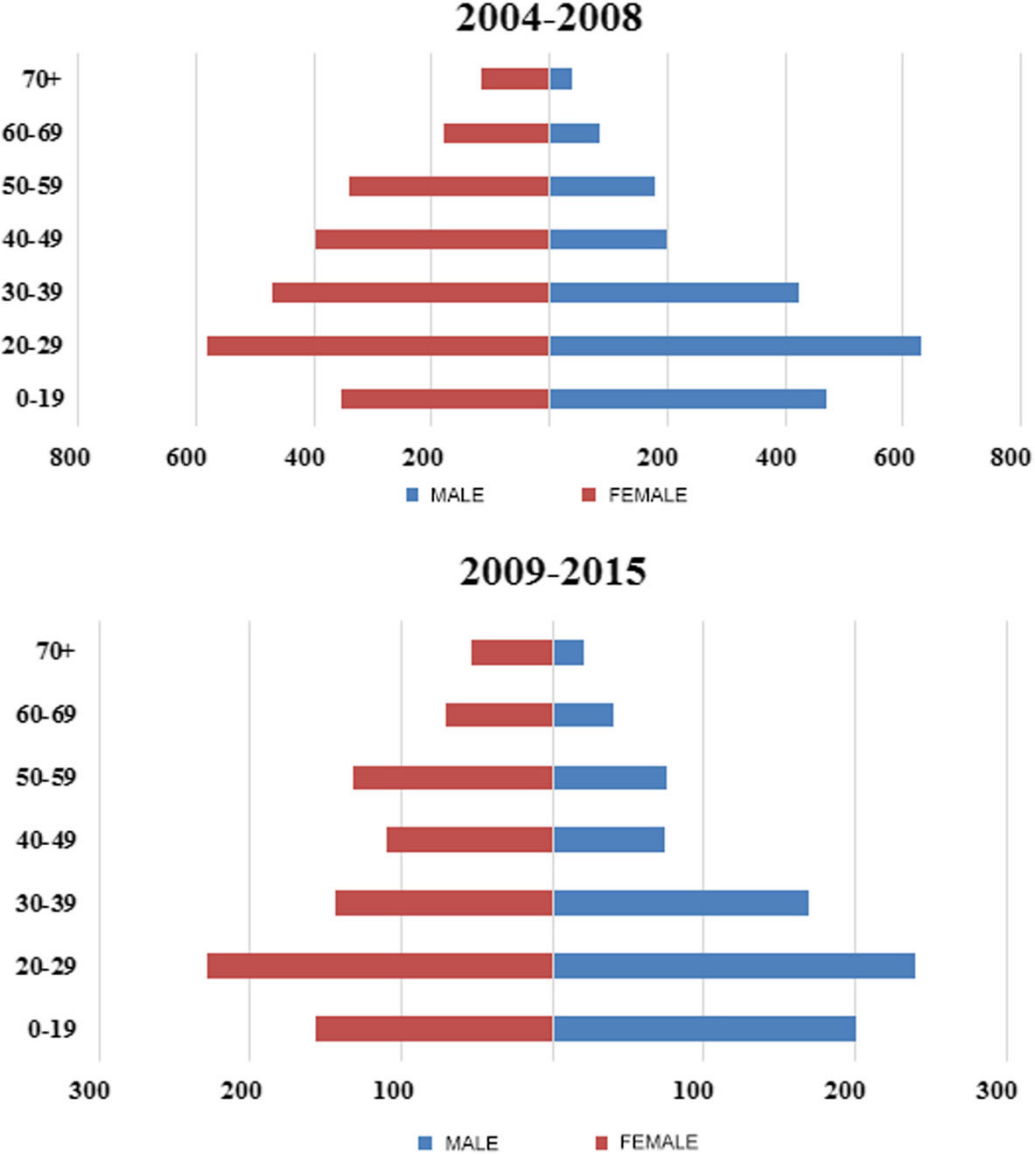
Age and gender distribution of newly Crohn’s disease patients diagnosed in 2004–2008/2009–2015

A total of 6173 (male/female: 46.12%/53.87%) newly diagnosed CD patients with physician-diagnosed Crohn’s disease were found between 2004 and 2015. 4458 and 1715 newly diagnosed CD patients with an available 3-year follow up period were identified between 01.01.2004 and 31.21.2008 and between 01.01.2009 and 01.01.2015. Distribution of age at diagnosis was similar in the two diagnostic eras, the most prevalent age group at the diagnosis of Crohn’s disease was the 20–29 years old group in both gender and in both diagnostic eras (Fig. 1).

### Therapeutic strategy

**Fig. 2:**
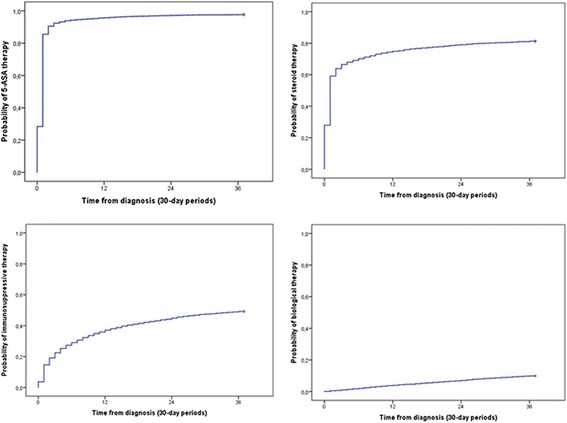
The cumulative probability of 5-ASA, steroid, IS and biological therapy in the whole observation period

Drug exposures were calculated in a time dependent analysis. The cumulative probability of 5-ASA, steroid, IS and biological therapy were 95.7, 74.8, 37.1 and 4% after 12 months and 97.7, 81.3, 49.4 and 9.9% after 3 years from diagnosis (Fig. 2).

The use of 5-ASA was 93%/90.8% after 3 months and 97.9%/97.1% after 3 years from the diagnosis in the whole patient groups diagnosed before and after 2009, and it did not differ according to the different maximal treatment steps. Use of 5-ASA was similar in the two diagnostic eras in each patient group.

**Fig. 3:**
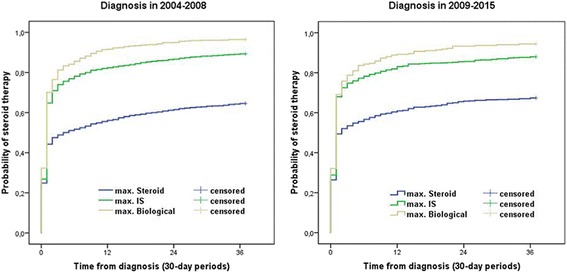
Probability of steroid therapy according to the maximum treatment steps

Cumulative steroid exposure was also comparable in both groups (64.4%/66% after 3 months, 72.6%/74.2% after 12 months, 79.8%/79.9% after 3 years in patients diagnosed before and after 2009). Probability of early steroid exposure was different according to the maximum treatment step but not according to the year of diagnosis: it was 48.9%/53.5% after the first 3 months from diagnosis in patients with steroids as maximum treatment step (pLogRank = 0.09), 74%/74.8% in patients with IS as maximum treatment step (pLogRank = 0.95) and 81.2%/78.7% in patients with biological therapy as maximum step (pLogRank = 0.31), in patients diagnosed before and after 2009 (Fig. 3).

**Fig. 4:**
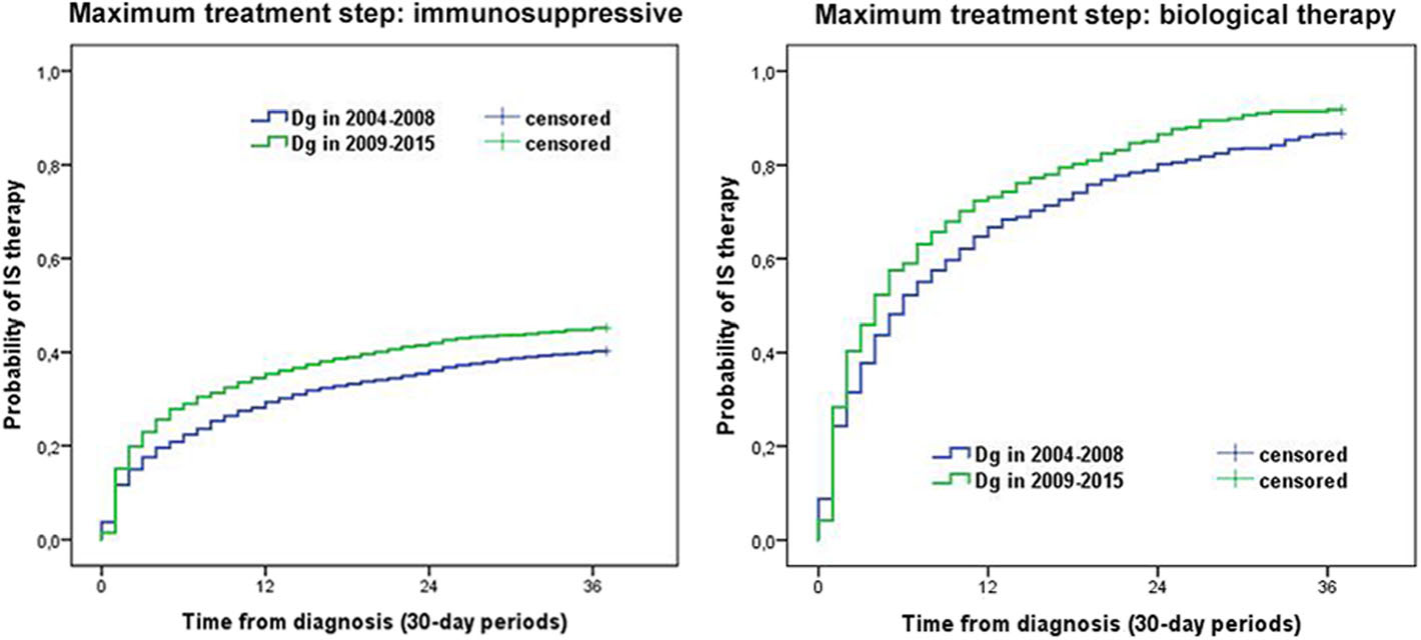
Probability of immunosuppressive therapy according to the diagnostic period and the maximal treatment steps

Exposure to immunosuppressive therapy was frequent in both diagnostic eras, with higher probability (pLogRank < 0.001 for the max. IS group and *p* = 0.007 for the max. Biological group) and earlier initiation in the second period. Probability of immunosuppressive therapy was 17.5% vs. 22.9% at 3 months, 29.3% vs. 35.4% at 1 year and 39.9% vs. 45.2% at 3 years in 2004–2008 vs. 2009–2015 in patients with IS as maximum treatment step. Exposure of IS was higher in patients needing anti-TNF therapy: 31.5% vs. 40.3% at 3 months, 66.7% vs. 73.1% at 1 year and 86.7% vs. 91.8% at 3 years in 2004–2008 vs. 2009–2015 (Fig. 4).

**Fig. 5:**
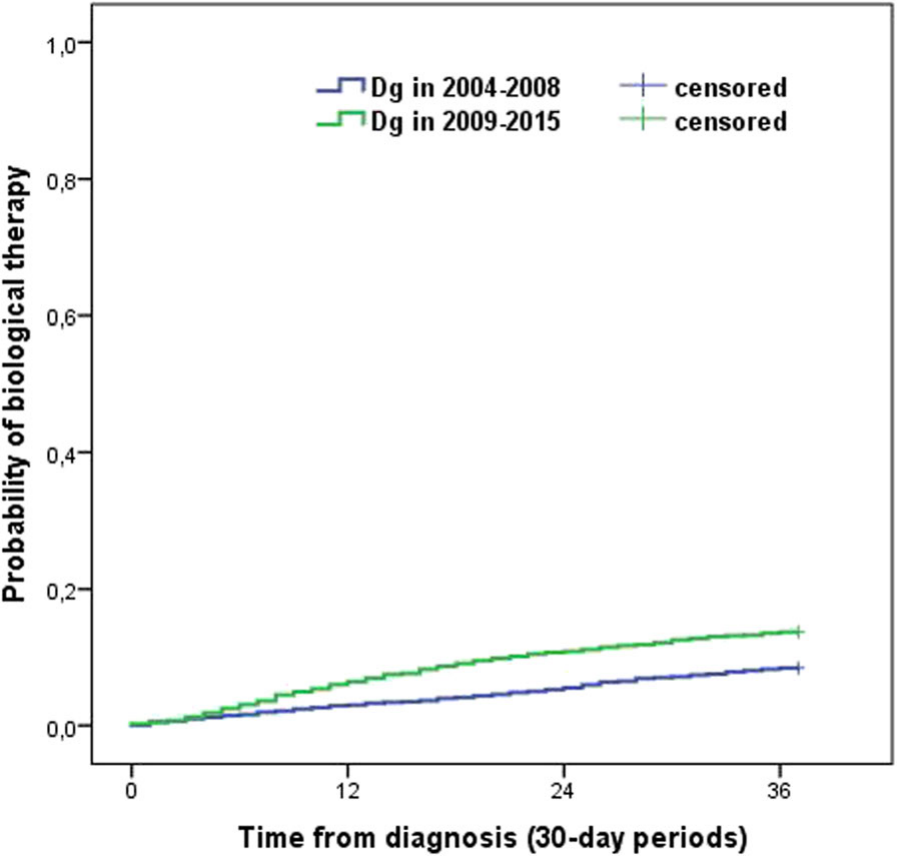
Probability of biological therapy according to the diagnostic period

The probability of biological therapy was significantly higher in patients diagnosed between 2009 and 2015 (pLogRank< 0.001). It was 1% vs. 1.2% after 3 months, 2.9% vs. 6.4% after 1 year and 8.4% vs. 13.7% after 3 years in 2004–2008 vs. 2009–2015 (Fig. 5).

### Hospitalizations and surgery rates according to the maximal treatment steps in the biological era

**Fig. 6:**
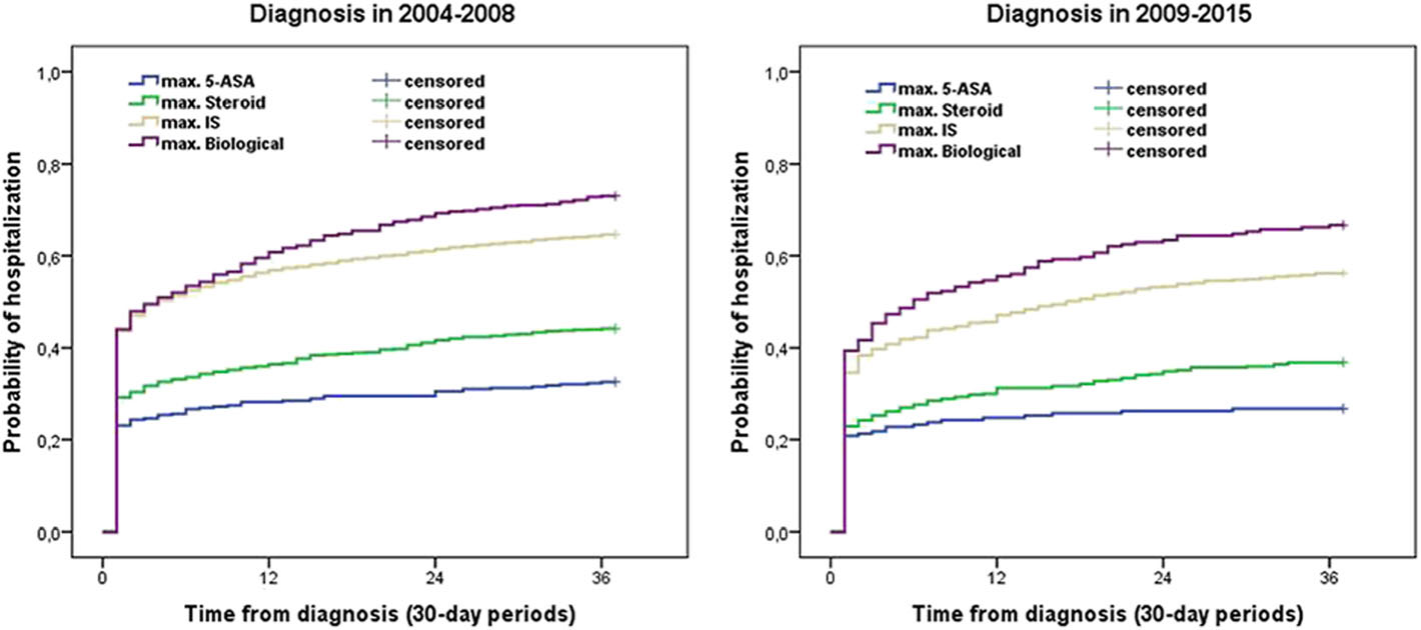
Probability of hospitalization according to the maximum treatment steps in patients diagnosed in 2004–2008/2009–2015

Probability of hospitalizations in the first 3-years after the diagnosis was significantly lower in patients diagnosed in 2009–2015 than in patients diagnosed in 2004–2008 (total probability: 55.7% vs. 47.4% in patients diagnosed in 2004–2008 vs. in 2009–2015, pLogRank < 0.001 for the total cohort). When we analysed the hospitalization rates according to the maximal treatment steps, the difference was significant in the steroid and IS group (total probabilities: 5-ASA group: 32.6% vs. 26.7% pLogRank = 0.16, Steroid group: 44.2% vs. 36.8% pLogRank = 0.007, IS group: 64.6% vs. 56.1% pLogRank< 0.001, Biological group: 73% vs. 66.7% pLogRank = 0.10) (Fig. 6).

**Fig. 7:**
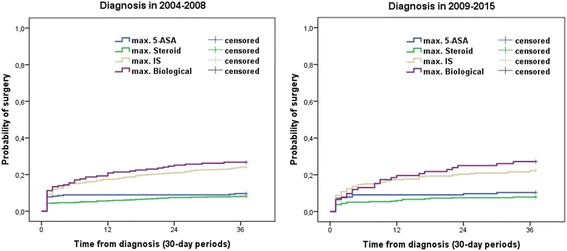
Probability of surgery according to the maximum treatment steps in patients diagnosed in 2004–2008/2009–2015

In contrast, surgery rates were altogether low and not significantly different in the two periods, but they were associated with the maximum therapeutic step. A proportion of patients underwent surgery at around the time of diagnosis (within 3 months from diagnosis) in both diagnostic eras, regardless of the maximum treatment step (9.1%/8.6% in the total cohort in 2004–2008/2009–2015). Thereafter the probability of surgery remained unchanged in patients with 5-ASA and steroid only, while it increased continuously in the IS and biological group (at 1 year: overall: 12% vs. 11.9%, biological therapy: 20.9% vs. 19.6%, IS: 17.4% vs. 17.3%, steroid 5.8% vs. 6%, 5-ASA 8.9% vs. 9.1%, at 3 years: overall: 16.0% vs. 15.3% (pLogRank = 0.672), biological therapy: 26,7% vs. 27.2% (pLogRank = 0.993), IS: 24.1% vs. 22.2% (pLogRank = 0.565), steroid 8.1% vs. 7.9% (pLogRank = 0.896), 5-ASA 10% vs. 11% (pLogRank = 0.816) in patients diagnosed in 2004–2008 vs. 2009–2015) (Fig. 7).

## Discussion

The aim of the present study was to demonstrate a nationwide description about the therapeutic strategy and outcomes in newly diagnosed patients with Crohn’s disease in the biological era in Hungary.

The main findings of the study were that hospitalization but not surgical rates differed significantly in patients diagnosed between 2004 and 2008 and between 2009 and 2015, along with the earlier and more frequent use of IS and biologicals in the second diagnostic period. Of note, surgery rates were altogether low. In addition, hospitalizations and surgeries were more frequent in the biological and the IS group compared to patients with steroids and 5-ASA only, suggesting that maximal therapeutic steps can be used as severity markers in patients with IBD when analysing health insurance databases.

In the present study, the overall probability of surgery was 9.1%/8.6% after 3 months, 12%/11.9% after 1 year and 16%/15.3% after 3 years from diagnosis among patients diagnosed in 2004–2008/2009–2015. This is in line with new data reported after 2000 [6]. According to the meta-analysis of Frolkis et al. based on 30 population-based studies, the cumulative risk of surgery in CD-patients diagnosed in the 2000s was 12.6 and 24.2% after 1 and 5 years from diagnosis [19]. The study from Manitoba also reported that probability of surgery was 10% and 18% after 1 and 5 year from diagnosis in CD patients diagnosed after 2000 [8]. In contrast, ECCO-EpiCom 2011 inception cohort study showed that 24 and 7% of patients from Eastern Europe and Western Europe/Australia underwent surgery within the first year of diagnosis [13].

Interestingly, the present study demonstrated that a stable proportion of patients (app. 9%) had surgery at around the diagnosis (within 3 months from diagnosis) in both diagnostic eras, independently from the maximum treatment step and further disease course. Similarly, Solberg et al. reported that very early CD-related surgery rates remained unchanged and did not influence long term clinical remission rates or outcomes [20]. In contrast, a recent study from Portugal concluded that patients undergoing an early surgery after diagnosis have an increased reoperation rate [21]. Of note, the rate of early surgery within 1 year from diagnosis remained stable in most epidemiological cohorts suggesting that at least a propostion of early surgeries can be regarded as unavoidable [6, 19].

Furthermore, our results suggest that the earlier and more frequent use of biological therapy did not reduce the probability of early surgery during the first 3 years from diagnosis. Previous population-based studies reported that the need for surgery significantly decreased between the 1980s and 2000s, parallel with the increased use of immunosuppressives [7, 8, 22]. Similarly, a former study from Hungary demonstrated that increased and earlier azathioprine use was independently associated with significant change in the natural history of CD and reduced surgery rates [17]. However, only few population-based studies are available from the biological era, and it is still conflictive whether biological therapy further decreases surgical rates on a population-based level [6, 23]. A recent meta-analysis based on 7 randomized controlled trial studies concluded that anti-TNFs were efficacious in reducing hospitalization and surgery rates compared to placebo, while no statistically significant reduction was noted to azathioprine (AZA) and vedolizumab in moderate-to-severe CD patients [23]. Similarly to our findings, a Spanish study concluded that IFX availability did not reduce surgical requirements with a step-up therapeutic algorithm in newly diagnosed CD patients [24]. Moreover, a study based on the Manitoba-cohort suggested that decline in surgery was associated with the care by a gastroenterology specialist but not with the immunomodulatory use in multivariate analysis. This study also concluded that use of more aggressive medical therapy can be a severity marker in non-randomized trial settings [8]. Furthermore, the recent update of the 5-years follow-up of the 2010 ECCO-EpiCom inception cohort study also reported that similar surgery and hospitalization rates were shown in Eastern and Western Europe, despite of more early and aggressive treatment with biologicals in Western Europe [25]. A study from Northern California concluded that prevalence of prolonged steroid exposure and hospitalization rates declined, whereas surgery rates remained constant in parallel with the increasing use of IS and biologicals in CD [26].

The probability of hospitalization was frequent around the diagnosis, but thereafter the frequency of hospitalization was not associated with the maximal therapeutic steps in the present study. Frequency of hospitalization was lower in all treatment groups in the second diagnostic period (decrease was significant in the steroid and IS group), showing a change in the patient management and the possible role of the accelerated treatment strategy. These results are in concordance with previous data that hospitalization is frequent around the diagnosis but it declines significantly from the second year after the diagnosis [27, 28]. Nevertheless, hospitalization rates vary significantly between countries, depending on health care/reimbursement policy and ethnic differences [2, 6].

The other primary aim of the study was to analyse the therapeutic strategy in the two diagnostic eras. Surprisingly, almost every CD patient received 5-ASA therapy in Hungary at around the diagnosis. This result is in concordance with the result of the ECCO-EpiCom 2011 inception cohort study and with other nationwide studies [13, 14]. Possible explanations are that physicians prescribe 5-ASAs until the diagnosis is finally confirmed or patients with mild CD are given 5-ASAs for maintenance therapy. Similarly, a national survey from Germany showed that app. 40% of physicians followed “wait and see” management strategy without any medical therapy in mild CD patients as maintenance, while third of physicians preferred 5-ASA in these cases [29].

Interestingly, the use of steroids remained common in both periods. The majority of patient received steroid therapy as initial treatment at diagnosis and it increased slightly over time. Compared to other recent population-based studies, the use of steroids was more frequent in the present cohort. In previous population-based studies the half of the CD patients received steroids during the early disease course [8, 30, 31]. Other studies showed steroid sparing effect of the increased IS and biological use [7, 26]. In contrast, the ECCO-EpiCom 2011 inception cohort reported that 61 and 67% of patients from Eastern Europe and Western Europe/Australia received systemic steroids during the first year of disease course [13].

The use of immunosuppressives was more frequent in the second period with earlier initiation. This suggests an accelerated step-care strategy in Hungary, similarly to those reported from the Spanish Administrative Database [24]. In the first period, the probability of IS use after 1 year from diagnosis was similar to the previous data from Hungary from the same decade (35.5% vs. 34.8%), while rate of IS use in the second period was higher, similar to rates from Western-Europe (41.3% vs. 45%) [13, 16, 32].

Finally, the use of biologicals was more frequent with earlier initiation in the second period in accordance with the changes of the drug prescription guidelines of OEP allowing an earlier and more extensive availability of anti-TNFs after 2009 in Hungary. In a previous study by Rencz et al. Hungary had the best access to biologicals in IBD in Central-Eastern Europe [33]. However, the use of biological therapy was in line with the Eastern European data in the 2010 ECCO EpiCom inception cohort study, it remained less frequent compared to Western Europe [25, 32].

The authors are aware of the possible limitations of the present work including the limitations of administrative databases such as the lack of disease phenotype data, time bias due to patient stratification or possible bias of patient ascertainment. In contrast, the strength of the present study is the nationwide nature of the study. The administrative database of the National Health Fund is unique in many regards, as in Hungary this is the only insurance plan with a full coverage and it collects in-, outpatient visits, diagnostic procedures and drug prescription data. Thus, it offers an invaluable opportunity to study treatment practices and changes in outcomes representative for the full spectrum of IBD patients in Hungary. Moreover, earlier publications suggested that the first 3-years of the disease are the most sensitive and representative for the further disease course [16, 34]. Thus, results from the present study can be extrapolated in the prediction of the further disease course as well as may be generalizable to most administrative databases from countries with similar health insurance policies.

## Conclusion

In conclusion, the present study, representing the nationwide practices in Hungary suggests, that there is an accelerated therapeutic strategy in CD in Hungary with IS and biological use becoming more frequent. Hospitalization decreased in the whole cohort, while surgery remained low but constant during the observation period.

Exposure to steroids and 5-ASA remained high. The association between maximal treatment step and hospitalization and surgery rates suggests that maximal treatment steps can be regarded as proxy severity markers in patients with IBD.

## Abbreviations

5-ASA: 5-Aminosalicylic Acid
Anti-TNF: Anti - tumor necrosis factor
AZA: Azathioprine
CD: Crohn’s disease
ECCO-EpiCom: European Crohn’s Colitis Organization Epidemiology Committee
IBD: Inflammatory bowel disease
ICD-10: 10th revision of the International Statistical Classification of Diseases and Related Health Problems
IS: Immunosuppressives,
OEP: National Health Insurance Fund,
